# Comparison of subsequent injury categorisation (SIC) models and their application in a sporting population

**DOI:** 10.1186/s40621-019-0183-1

**Published:** 2019-03-11

**Authors:** Liam A. Toohey, Michael K. Drew, Lauren V. Fortington, Miranda J. Menaspa, Caroline F. Finch, Jill L. Cook

**Affiliations:** 1 La Trobe Sport and Exercise Medicine Research Centre, La Trobe University, School of Allied Health (Physiotherapy), Bundoora, VIC 3086 Australia; 2 Athlete Availability Program, Applied Technology and Innovation, Australian Institute of Sport, Leverrier Street, Bruce, ACT 2617 Australia; 3 Physical Therapies, Australian Institute of Sport, Bruce, ACT Australia; 4 School of Medical and Health Sciences, Edith Cowan University, Joondalup, WA Australia; 5 Federation University Australia, Ballarat, Australia

**Keywords:** Sports injury, Athletic injury, Injury classification, Injury definition, Sports medicine, Epidemiology, Water sports, Water polo

## Abstract

**Background:**

The original subsequent injury categorisation (SIC-1.0) model aimed to classify relationships between chronological injury sequences to provide insight into the complexity and causation of subsequent injury occurrence. An updated model has recently been published. Comparison of the data coded according to the original and revised subsequent injury categorisation (SIC-1.0 and SIC-2.0) models has yet been formally compared.

**Methods:**

Medical attention injury data was prospectively collected for 42 elite water polo players over an 8 month surveillance period. The SIC-1.0 and SIC-2.0 models were retrospectively applied to the injury data. The injury categorisation from the two models was compared using descriptive statistics.

**Results:**

Seventy-four injuries were sustained by the 42 players (median = 2, range = 0–5), of which 32 injuries (43.2%) occurred subsequent to a previous injury. The majority of subsequent injuries were coded as occurring at a different site and being of a different nature, while also being considered clinically unrelated to the previous injury (SIC-1.0 category 10 = 57.9%; SIC-2.0 clinical category 16 = 54.4%). Application of the SIC-2.0 model resulted in a greater distribution of category allocation compared to the SIC-1.0 model that reflects a greater precision in the SIC-2.0 model.

**Conclusions:**

Subsequent injury categorisation of sport injury data can be undertaken using either the original (SIC-1.0) or the revised (SIC-2.0) model to obtain similar results. However, the SIC-2.0 model offers the ability to identify a larger number of mutually exclusive categories, while not relying on clinical adjudication for category allocation. The increased precision of SIC-2.0 is advantageous for clinical application and consideration of injury relationships.

**Electronic supplementary material:**

The online version of this article (10.1186/s40621-019-0183-1) contains supplementary material, which is available to authorized users.

## Background

Subsequent injuries, defined as any injury that occurs at any stage following an initial (index) injury, account for a large proportion of all injuries that are sustained within sporting populations. (Finch et al. 2017; Fortington et al. 2017) Understanding the within-person dependency of injuries is an important component of developing injury prevention and treatment strategies for athletes. (Finch and Cook 2014; Toohey et al. 2018) Meaningful consideration of the relationships underpinning subsequent injuries currently relies on categorisation frameworks that support the identification of how injury types are related in terms of body part, nature and side of injury.

The original subsequent injury categorisation (SIC-1.0) model (Finch and Cook 2014) provided ten mutually exclusive categories that extended beyond the limitations of previous classification models. One of the advancements provided by the SIC-1.0 model was the ability to distinguish the injury onset (acute or gradual) for subsequent injuries that were sustained at the same body site and nature to a previous injury. A second iteration of this model (SIC-2.0) has recently been published, (Toohey et al. 2018) which provides a two-tiered hierarchical structure for subsequent injury categorisation (Table 1). The application of the SIC-2.0 overarching data-driven level of categorisation has already been demonstrated in a sporting dataset, (Toohey et al. 2018) but the application of sub-categorisation to the clinical level of injury relatedness that the model offers has not been demonstrated. Nor has there been an evaluation between the two models’ (SIC-1.0 and SIC-2.0) classification outputs, which currently limits the ability to make comparisons between the two. Thus, the aims of this study were to: (1) apply the revised SIC-2.0 model to a sporting dataset to both the data-driven and clinical sub-categorisation levels and to (2) compare the categorisation output with the original SIC-1.0 model output within the same sporting injury dataset.

**Table 1:** Comparison of the original (SIC-1.0) and revised (SIC-2.0) subsequent injury categorisation models

SIC-2.0 data-driven category (Toohey et al. 2018)	SIC-2.0 clinical category (Toohey et al. 2018)	Category description	SIC-1.0 category (Finch and Cook 2014)
I	1	No subsequent injury; only one injury was sustained by the athlete throughout the surveillance period	1
II	2	Re-injury after recovery, to the same site, same nature, same side, and same structure (related)	2^a^
	3	Re-injury after recovery, to the same site, same nature, same side, and same structure (unrelated)	6^a^
III	4	Acute exacerbation before recovery, to the same site, same nature, same side, and same structure	3^a^
	5	Continual/sporadic exacerbation before recovery, to the same site, same nature, same side, and same structure (related)	4^a^
	6	Continual/sporadic exacerbation before recovery, to the same site, same nature, same side, and same structure (unrelated)	5^a^
IV	7	Injury to the same site, same nature, same side, but of a different structure (related)	2-6^a^
	8	Injury to the same site, same nature, same side, but of a different structure (unrelated)	2-6^a^
V	9	Injury to the same site, same nature, but different side (related)	2-6^a^
	10	Injury to the same site, same nature, but different side (unrelated)	2-6^a^
VI	11	Injury to the same site but of a different nature (related)	7
	12	Injury to the same site but of a different nature (unrelated)	8
VII	13	Injury to a different site, but of the same nature (related)	9^b^
	14	Injury to a different site, but of the same nature (unrelated)	10^b^
VIII	15	Injury to a different site and of a different nature (related)	9^b^
	16	Injury to a different site and of a different nature (unrelated)	10^b^

Adapted from Toohey et al., 2018 (Toohey et al. 2018) with permission

^a^ side and structure of injury was not differentiated in the SIC-1.0 model; ^b^ injury nature at different site was not differentiated in the SIC-1.0 model

## Methods

The classification outputs from both the SIC-1.0 and SIC-2.0 models were generated based on prospectively collected injury data for 42 elite water polo players (36 women, mean age: 19.9 ± 3.4; 6 men, mean age: 20.8 ± 4.1) over eight consecutive months (August 2013 to March 2014).

All injury data were entered into a centralised database (Athlete Management System (AMS), Fusion Sport Pty Ltd., Brisbane, Australia) by the squad’s senior sports physiotherapist (MJM). A four character Orchard Sports Injury Classification System 10 (OSICS-10.1) (Rae and Orchard 2007) injury diagnosis code was assigned prospectively at the time of injury or treatment to each injury by the physiotherapist (MJM), with the side of injury occurrence, mechanism of injury, date of injury, date of return to training, and date of full injury resolution also recorded. All data were de-identified (but linked by a unique athlete ID) and injuries were time ordered according to the date of injury for each injured athlete. (Finch and Fortington 2018)

For SIC-1.0, following the 8 month surveillance period, the injury data were retrospectively coded using the SIC-1.0 model (Finch and Cook 2014), again by the same physiotherapist (MJM). (Wallis and Drew 2014) For SIC-2.0, the injury data were retrospectively coded using the updated model, (Toohey et al. 2018) to both the data-driven and clinical category levels by one author, independent to the water polo squad (LAT). For consistency, the sub-categorisation of the data-driven categories reached in the SIC-2.0 model was performed utilising the original clinical decision that determined whether or not a subsequent injury was determined to be clinically related to a previous injury as determined in the SIC-1.0 model categorisation process. This decision was used to ensure consistency between model comparisons. A descriptive analysis is presented to compare the output of the two models by the number and percentage within each category. Ethical approval for the study was obtained from the Human Research Ethics Committees of the Australian Institute of Sport and La Trobe University (Approval Number 20160401).

## Results

A total of 74 injuries were sustained by 42 athletes over the surveillance period (median = 2, range = 0–5). Thirty-two (43.2%) injuries were subsequent to a previous injury within the surveillance period. Injuries to the shoulder (*n* = 17, 23.0%), elbow (*n* = 12, 16.2%) and lumbar spine (*n* = 10, 13.5%) were the most frequent according to body site. The most common type of injuries sustained were impingement (*n* = 16, 21.6%), joint sprains (*n* = 14, 18.2%) and muscle injuries (*n* = 12, 16.2%).

**Fig. 1:**
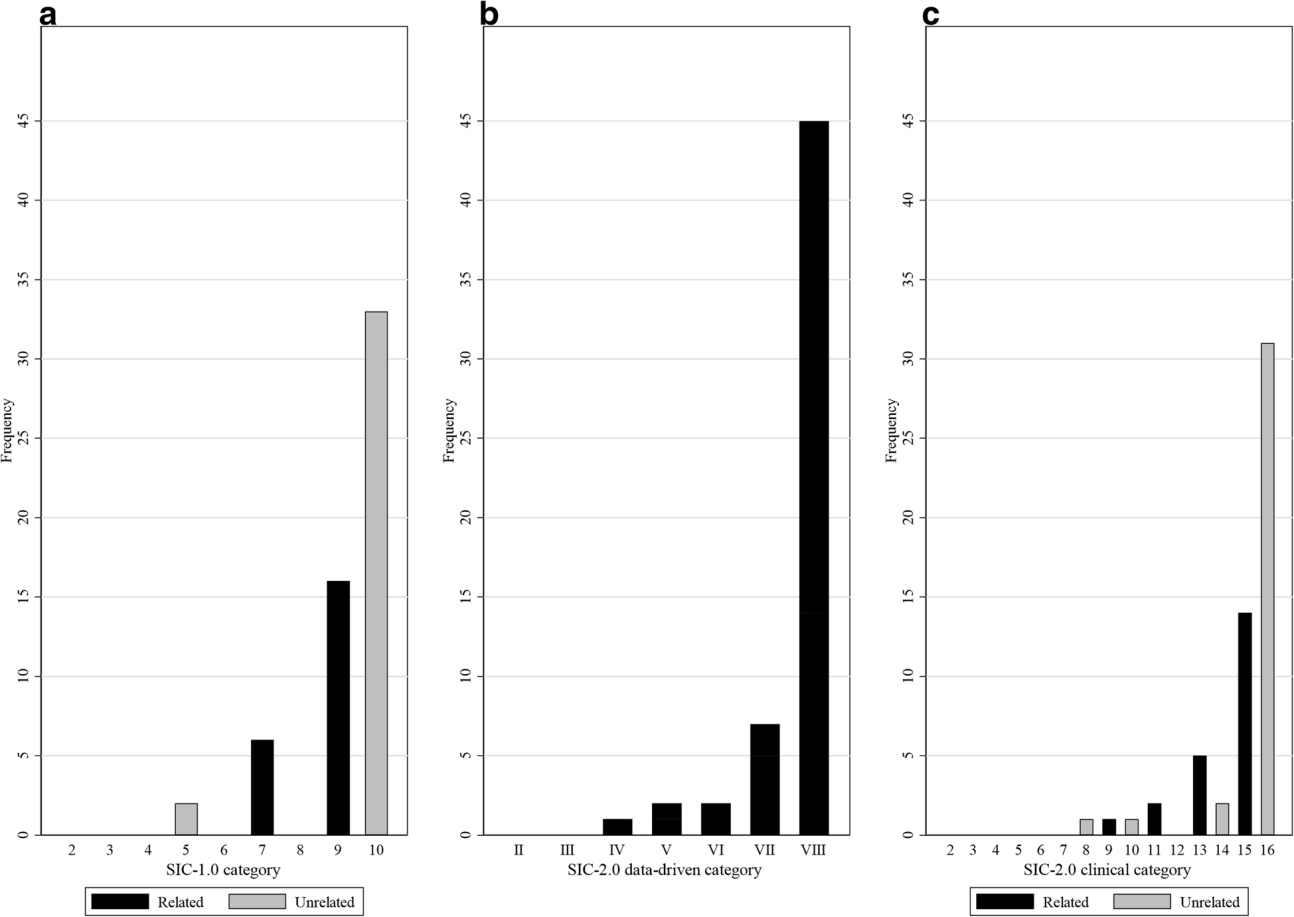
Subsequent injury categorisation output from the original subsequent injury categorisation (SIC-1.0) model (**a**) and the revised subsequent injury categorisation (SIC-2.0) model at the data-driven level of categorisation (**b**) and the clinical level of categorisation (**c**)

From the SIC-1.0 model, the majority of subsequent injury relationships were categorised as category 10 injuries (57.9%; injury to a different site and different nature and unrelated to the previous injury/injuries), or category 9 injuries (28.1%; injury to a different site and different nature and related to the previous injury/injuries). Of the ten SIC-1.0 categories, only four (categories 5, 7, 9, and 10) were identified during the categorisation process. In total, 38.6% of the subsequent injuries were determined to be clinically related to a previous injury (Fig. 1a) (Additional file 1).

Categorisation to the data-driven level of the SIC-2.0 model found category VIII (injury to a different site and of a different nature) subsequent injury relationships to be the most common (79.3%), followed by category VII (12.1%; injuries to a different site, but of the same nature) (Fig. 1b) (Additional file 1). Sub-categorisation to the clinical level of the SIC-2.0 model, identified SIC-2.0 categories 16 (54.4%; injury to a different site and of a different nature and unrelated) and 15 (24.6%; injury to a different site and of a different nature and related) to be the most commonly allocated categorisation codes (Fig. 1c) (Additional file 1).

**Fig. 2:**
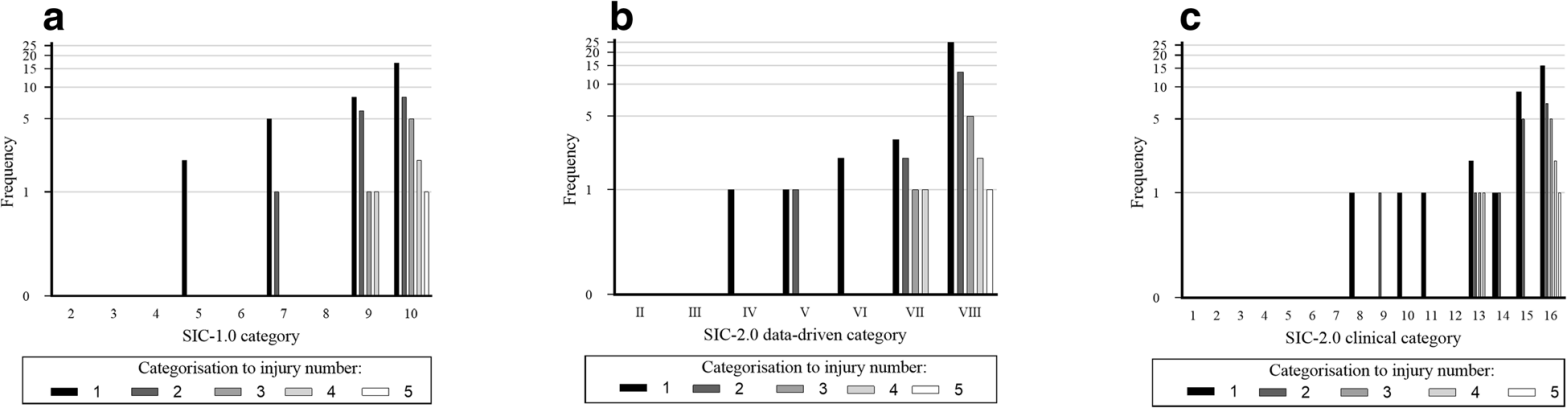
Application of the SIC-1.0 model (**a**), data-driven level of SIC-2.0 (**b**) and clinical level of categorisation (**c**) according to the injury number in order of temporal occurrence (Finch et al. 2017; Fortington et al. 2017; Finch and Cook 2014; Toohey et al. 2018; Rae and Orchard 2007)

When the category allocation was specified to the injuries according to the sequence that they were sustained in (i.e. 1st injury, 2nd injury, 3rd injury, 4th injury, or 5th injury), it was observed that the most common type of subsequent injury relationship identified by the different models was a different site and different nature (SIC-1.0 category 10 and 9; SIC-2.0 data driven category VIII and SIC-2.0 clinical category 16 and 15) (Fig. 2). Following the second injury being sustained, all of the subsequent injury relationships were identified to be of a different site and different nature.

## Discussion

This is the first paper to apply the SIC-2.0 model to the clinical sub-categorisation level, considering the influence that a clinical decision has on the segregation of subsequent injury categorisation. The application of the SIC-2.0 model to this level demonstrates the model’s ability to identify differences between the data-driven categories.

This study also shows that similar categorisation results are observed when the SIC-1.0 and SIC-2.0 models are applied to the same dataset. The most common relationship between injuries within a time-series categorised them as being of a different site and different nature. This finding is consistent with studies across a range of sports using both the SIC-1.0 model (Finch et al. 2017; Finch and Cook 2014; Finch et al. 2015; Moore et al. 2018) and the SIC-2.0 model. (Toohey et al. 2018) The ability of the SIC-2.0 model to identify and differentiate a larger number of different relationships between a subsequent injury and the injuries that preceded it appears to offer a greater sensitivity for more precise categorisation than the SIC-1.0 model.

Considering the range of criteria that can be used define a subsequent injury as being clinically-related to a previous injury presents a complex question for sports medicine professionals. There are no established guidelines that outline what criteria should be considered when adjudicating whether a subsequent injury is related or not to a previous injury, (Toohey et al. 2018) rather research to date has replied on clinical reasoning to determine these relationships. (Finch et al. 2017; Finch and Cook 2014; Moore et al. 2018) The inter-rater reliability of the SIC-1.0 model, which requires the user to make a decision on injury relatedness, has been demonstrated to be strong between team clinicians and moderate to strong between a team clinician and a non-team clinician. (Moore et al. 2018) The level of agreement between raters reduces however when a clinician’s categorisation output is compared to that of a non-clinician rater. (Moore et al. 2018)

We did not attempt to define these criteria within this study, rather we chose to use an injury dataset that had previously been coded according to the SIC-1.0 model with the team clinician’s interpretation of what constituted an injury to be related. There are many potential factors that can be considered by a clinician on whether an injury is or is not related to a previous injury. (Moore et al. 2018) These include, but are not limited to: anatomical considerations (the body site, tissue type, and the side of injury), the time between injury occurrences, the biomechanical relationships between body segments specific to the demands of the sport, the established training workload following a previous injury, and residual deficits or changes in technique related to a previous injury, or potential changes in psychological status following a previous injury which could all influence the risk of sustaining a future injury.

To overcome the challenge of determining relatedness between injuries, at least until there is international agreement on how this is defined, a stronger focus on the data-driven categorisation of SIC-2.0 is recommended. The SIC-2.0 model was designed to offer an overarching data-driven approach to avoid the need for a clinical decision to be made in the categorisation process. This provides a standardised method of categorisation that is reproducible and the automated ability of the model provides a method free from human error. (Toohey et al. 2018) This is evidenced by the 100% inter-rater reliability between two physiotherapists and between the physiotherapists and the automated coding script. (Toohey et al. 2018) Concurrently, outcomes that rely on a subjective clinical decision to be made in the categorisation process, such as those produced at the clinical categorisation level of the SIC-2.0 model and the SIC-1.0 model, should be considered with some caution and ideally be determined at the time of injury, not retrospectively. It is acknowledged that an adjudicated determination of clinical relatedness between temporal injury sequences may provide additional valuable information for understanding subsequent injury occurrence. However, further research to determine the most appropriate criteria to define injury relatedness is required to achieve reliable categorisation output.

Application of the SIC-2.0 data-driven categorisation in this study has demonstrated, that even within a small dataset, a larger distribution of category allocation is achieved as a greater degree of precision in category allocation is possible when compared to the original SIC-1.0 model.

In this study, all of the subsequent injuries that followed a second injury within a temporal series occurred at a different site to previous injuries. This finding has implications for clinical rehabilitation which challenges the preliminary goal of restoring function of the specific injured site to prevent a recurrent injury to the same site. Medical staff also need to be aware that on return to play an athlete is at risk of sustaining another injury at a different site and rehabilitation should incorporate tertiary prevention strategies to mitigate the risk of different types of subsequent injury occurring. (Blanch and Gabbett 2016; Jacobsson and Timpka 2015; Toohey et al. 2017).

Subsequent injury categorisation offers sports injury researchers the ability to consider injury relationships beyond recurrent-only injuries, which have been demonstrated in numerous sports to only account for a very small proportion of all injuries sustained. (Finch et al. 2017; Toohey et al. 2018; Moore et al. 2018) Consideration of all possible inter-injury relationships provides a greater insight into the associations and an opportunity to investigate the mechanisms that underpin subsequent injury occurrence. (Shrier and Steele 2014) Through greater understanding, more specifically targeted tertiary injury prevention strategies can be developed for athletes who have already sustained an injury to help mitigate the risk of the subsequent injury types that are most likely to sequentially occur.

## Conclusion

The categorisation output of subsequent injury data in sport utilising the original (SIC-1.0) and revised (SIC-2.0) models offer comparable results. The ability of the SIC-2.0 model to execute the categorisation process without the reliance on clinical adjudication offers greater reliability and also allows non-clinicians to use the model accurately. The SIC-2.0 model provides a larger number of mutually exclusive categories, which enhances the precision of subsequent injury categorisation and enables improved analysis of injury relationships.

## Additional file

Additional file 1: Categorisation output distribution of the SIC-1.0 and SIC-2.0 models. (DOCX 18 kb)

## Abbreviations

AMS: Athlete management system
ID: Identification number
OSICS: Orchard sports injury coding system
SIC: Subsequent injury categorisation
SIC-1.0: Subsequent injury categorisation model (original version)
SIC-2.0: Subsequent injury categorisation model (revised version)
