# Supplementary material for: Comparison of subsequent injury categorisation (SIC) models and their application in a sporting population
Source: Inj Epidemiol. 2019 Mar 11;6:9. doi: 10.1186/s40621-019-0183-1 (PMC6582673; doi:10.1186/s40621-019-0183-1)
Supplement: Supplementary file 1 — Categorisation output distribution of the SIC-1.0 and SIC-2.0 models. (DOCX 18 kb) [file 40621_2019_183_MOESM1_ESM.docx]

| **Category description** | **SIC-1.0** | | **SIC-2.0 data-driven** | | **SIC-2.0 clinical** | |  |
| --- | --- | --- | --- | --- | --- | --- | --- |
|  | ***Category*** | **%** | ***Category*** | **%** | ***Category*** | **%** |  |
| No subsequent injury; only one injury was sustained by the athlete throughout the surveillance period | *1* | 0 | *I* | 0 | *1* | 0 |  |
| Re-injury after recovery, to the same site, same nature, same side, and same structure (related) | *2^a^* | 0 | *II* | 0 | *2* | 0 |  |
| Re-injury after recovery, to the same site, same nature, same side, and same structure (unrelated) | *6^a^* | 0 |  | - | *3* | 0 |  |
| Acute exacerbation before recovery, to the same site, same nature, same side, and same structure | *3^a^* | 0 | *III* | 0 | *4* | 0 |  |
| Continual/sporadic exacerbation before recovery, to the same site, same nature, same side, and same structure (related) | *4^a^* | 0 |  | - | *5* | 0 |  |
| Continual/sporadic exacerbation before recovery, to the same site, same nature, same side, and same structure (unrelated) | *5^a^* | 3.5 |  | - | *6* | 0 |  |
| Injury to the same site, same nature, same side, but of a different structure (related) | *2-6^a^* | 0 | *IV* | 1.7 | *7* | 0 |  |
| Injury to the same site, same nature, same side, but of a different structure (unrelated) | *2-6^a^* | 0 |  | - | *8* | 1.7 |  |
| Injury to the same site, same nature, but different side (related) | *2-6^a^* | 0 | *V* | 3.4 | *9* | 1.7 |  |
| Injury to the same site, same nature, but different side (unrelated) | *2-6^a^* | 0 |  | - | *10* | 1.7 |  |
| Injury to the same site but of a different nature (related) | *7* | 10.5 | *VI* | 3.4 | *11* | 3.5 |  |
| Injury to the same site but of a different nature (unrelated) | *8* | 0 |  | - | *12* | 0 |  |
| Injury to a different site, but of the same nature (related) | *9^b^* | 28.1^c^ | *VII* | 12.1 | *13* | 8.8 |  |
| Injury to a different site, but of the same nature (unrelated) | *10^b^* | 57.9^c^ |  | - | *14* | 3.5 |  |
| Injury to a different site and of a different nature (related) | *9^b^* | 28.1^c^ | *VIII* | 79.3 | *15* | 24.6 |  |
| Injury to a different site and of a different nature (unrelated) | *10^b^* | 57.9^c^ |  | - | *16* | 54.4 |  |

**Additional file 1.**

^a^ side and structure of injury was not differentiated in the SIC-1.0 model; ^b^ injury nature at different site was not differentiated in the SIC-1.0 model; ^c^ SIC-1.0 categories 9 and 10 presented twice within table

Adapted from Toohey et al., 2018(4) with permission
